# Phenylbutyrate improves recognition memory and increases dendritic spines in the prefrontal cortex and hippocampus in hypertensive female rats

**DOI:** 10.1007/s00429-026-03216-3

**Published:** 2026-11-10

**Authors:** Edwin Martínez-Vásquez, Leonardo Aguilar-Hernández, Ma de Jesús Gómez-Villalobos, Eduardo Baltazar-Gaytan, Gonzalo Flores, Julio Cesar Morales-Medina

**Affiliations:** 1 Instituto de Fisiología, Benemérita Universidad Autónoma de Puebla (BUAP), 14 Sur 6301, 72570 Puebla, Mexico; 2 Centro de Investigación en Reproducción Animal, Cinvestav-Universidad Autónoma de Tlaxcala, 90128 Tlaxcala, Mexico; 3 Facultad de Medicina, Universidad Veracruzana (UV) Región Córdoba – Orizaba, Campus Ciudad Mendoza, 94740 Veracruz Orizaba, Mexico

**Keywords:** Dorsal hippocampus, Phenylbutyrate, Prefrontal cortex, Recognition memory, Spine morphology, Spontaneously hypertensive (SH) rats

## Abstract

Hypertension and aging are two major global health challenges. They frequently coexist, presenting with aging-related neuronal alterations which are exacerbated under hypertensive conditions. In the present study, we utilized female, 12-month-old spontaneously hypertensive (SH) rats as a translational animal model of combined hypertension and aging. Phenylbutyrate (PB), a compound with reported anti-inflammatory and neuroprotective properties was evaluated for its effects on recognition memory and dendritic architecture. PB treatment significantly improved both short- and long-term recognition memory, as assessed by the novel object recognition test. At the structural level, PB increased dendritic spine density in the prefrontal cortex (PFC) and in multiple subregions of the dorsal hippocampus, including CA1, CA3, and the dentate gyrus. Moreover, PB selectively enhanced the proportion of mature spine phenotypes, increasing mushroom and bifurcated spines in the PFC and hippocampal subregions examined. Together, these findings indicate that PB mitigates recognition memory decline and preserves dendritic spine density and morphology in cortico-hippocampal circuits in this animal model. Our results support PB as a potential therapeutic strategy for neurocognitive dysfunction associated with the comorbidity of hypertension and aging.

## Introduction

According to the World Health Organization (WHO), in 2024 an estimated 1.4 billion adults aged 30–79 years worldwide had hypertension (blood pressure ≥ 140/90 mmHg) — representing approximately 33% of adults in this age range globally (Iqbal and Jamal 2025; WHO, 2025). Development of hypertension is gradual but continuous, however, symptoms do not usually arrive until long after tissue damage has begun (Brouwers et al. 2021). Brain, kidneys and heart are the main organs affected as they are susceptible to endothelial and constant elevated pressure damage (Canavan and O’Donnell 2022; Mavrogeni et al. 2022). In hypertensive subjects, ischemic stroke, intracerebral hemorrhage, hypertensive encephalopathy and vascular dementia are often observed (Santisteban et al. 2023; Ungvari et al. 2021; Wajngarten and Silva 2019). The constant elevated pressure leads to damage in capillaries and small arterioles, inducing microvascular damage (Baggeroer et al. 2024), brain barrier rupture (Katsi et al. 2020) and chronic neuroinflammation (Bajwa and Klegeris 2022), leading to neuronal and glial damage and finally to cognitive decline and dementia (Li et al., 2016; Schiffrin and Engert 2023). Some studies have indicated the neuronal damage and cognitive alterations appear approximately ≥ 20 years after the onset of hypertension or in patients ≥ 50 years old (Li i et al. 2022, 2024; Walker et al. 2019). Moreover, the progression of these alterations may be further accelerated by the tissue and cellular environment associated with normal aging (Oliveros et al. 2020). Finally, life expectancy in hypertensive patients is shorter than normotensive subjects (Franco et al. 2005; Zhang et al. 2010).

Normal aging is characterized by the continuous accumulation of cellular damage and waste (Gladyshev et al. 2021), accompanied by progressive morphological alterations and global functional decline (Chalise 2019; Khan et al. 2017). In the aged brain, there are anatomical changes, including global reduction in volume and weight, thinning of the gray and white matter and bigger cerebral sulci and ventricles (Irimia 2021; Jin et al. 2018). In neurons, a reduction in synaptic plasticity and loss of dendritic spines is observed (Barrantes 2024; Flores et al. 2025; Huang et al. 2020). These alterations are prominent in the prefrontal cortex (PFC), hippocampus and temporal lobe (Bettio et al. 2017; Raz et al. 2005; Raz and Rodrigue 2006), and are associated with underlying inflammation, senescence, and oxidative stress (Aguilar-Hernández et al. 2023; Jurcau et al. 2024; Pole et al. 2016; Sikora et al. 2021). Functionally, there is an increase in the risk of cognitive decline (Gonzales et al. 2022). Indeed, cognitive decline complications are more severe in aged female patients with chronic uncontrolled hypertension than in male subjects (Kaur et al. 2025; Koran et al. 2017; Levine et al. 2021; Waldstein and Katzel 2004; Wood Alexander et al. 2025).

Phenylbutyrate (PB) is a molecule approved by the FDA for use in urea cycle disorders as it enhances nitrogen elimination and the detoxification of ammonium (FDA, 1996, 2013). However, preclinical studies have revealed other therapeutical properties of PB such as anti-inflammation, epigenetic modulation, improvement of mitochondrial performance, endothelial function, and neuroprotection (Carlisle et al. 2016; Jangra et al. 2016; Juarez et al. 2024; Rivera et al. 2024). In this study, we use spontaneously hypertensive (SH) rat strain as model of elevated arterial pressure. While several studies have looked at neuronal and cognitive changes in this animal model, most of them were carried out in male rats (Brocca et al. 2013; Johnson et al. 2020; Meneses et al. 1996; Nakamura-Palacios et al. 1996; Sánchez et al. 2011; Shih et al. 2016; Sontag et al. 2013; Wyss et al. 1992). Here, we use female rats to determine whether PB is capable of reducing the dendritic spine and memory alterations induced by hypertension and aging in female rats.

## Materials and methods

### Animals and treatment

24 SH rats at 12 months-old were acquired from the Claude Bernard bioterio of the Benemérita Universidad Autónoma de Puebla, Puebla. They were maintained in groups of 3–4 in acrylic boxes from the time of their acquisition. In addition, rats were kept under standard conditions of temperature (24 °C), humidity, dark/light cycle of 12 h and food/water *ad libitum*. All handling of rodents was carried out in accordance with the guidelines of NOM-062-ZOO (1999) Official Mexican Standard and the internal regulations of the animal facility under the FLAG-UALVIEP-17-1 committee. Sample size was selected based on previous studies evaluating dendritic spine density and behavioral outcomes in rodent models (Aguilar-Hernández et al. 2026; Monroy et al. 2025), which commonly employed 6–12 animals per experimental group and successfully detected treatment-induced morphological changes.

### Blood pressure determination

Blood pressure (BP) was measured in each rat one day before treatment using a non-invasive tail-cuff method targeting the caudal artery as reported previously (Coatl-Cuaya, et al., 2022). Animals were acclimatized one day before the measurement to reduce stress-induced variability. Next, rats were placed in the measurement environment one hour before the procedure. Each rat was then positioned in a restraint device for 5 min, then a tail-cuff device (XBP1001 Rat Tail Blood Pressure System, Kent Scientific Corporation, Connecticut, USA) was placed at the base of the tail and inflated to 200–250 mmHg. The cuff was gradually deflated, and a volume pressure recording-sensor detected the return of blood flow, allowing the determination of systolic and diastolic BP. 10 consecutive measurements were performed for each animal, and the average of stable readings was determined.

### Treatment

Animals were randomly distributed in two groups, one of which received 20 mg/kg of PB (Suzhou Leah New Material Technology Co., Ltd., Suzhou, China) prepared in physiological solution, while the control group received a similar volume of physiological solution, vehicle (VH) (Hartmann solution, PiSA Farmaceutica, México) (Carvajal-Flores et al. 2020). PB was administered via intraperitoneal injection daily for 12 weeks. The dose and route of PB administration was based on the proven plasticity effects in the previous study of our group (Carvajal-Flores et al. 2020). All animals used in the project belong to a single cohort; thus, environmental and experimenter handling differences were homogeneous for each rodent.

### Behavioral test

**Fig. 1:**
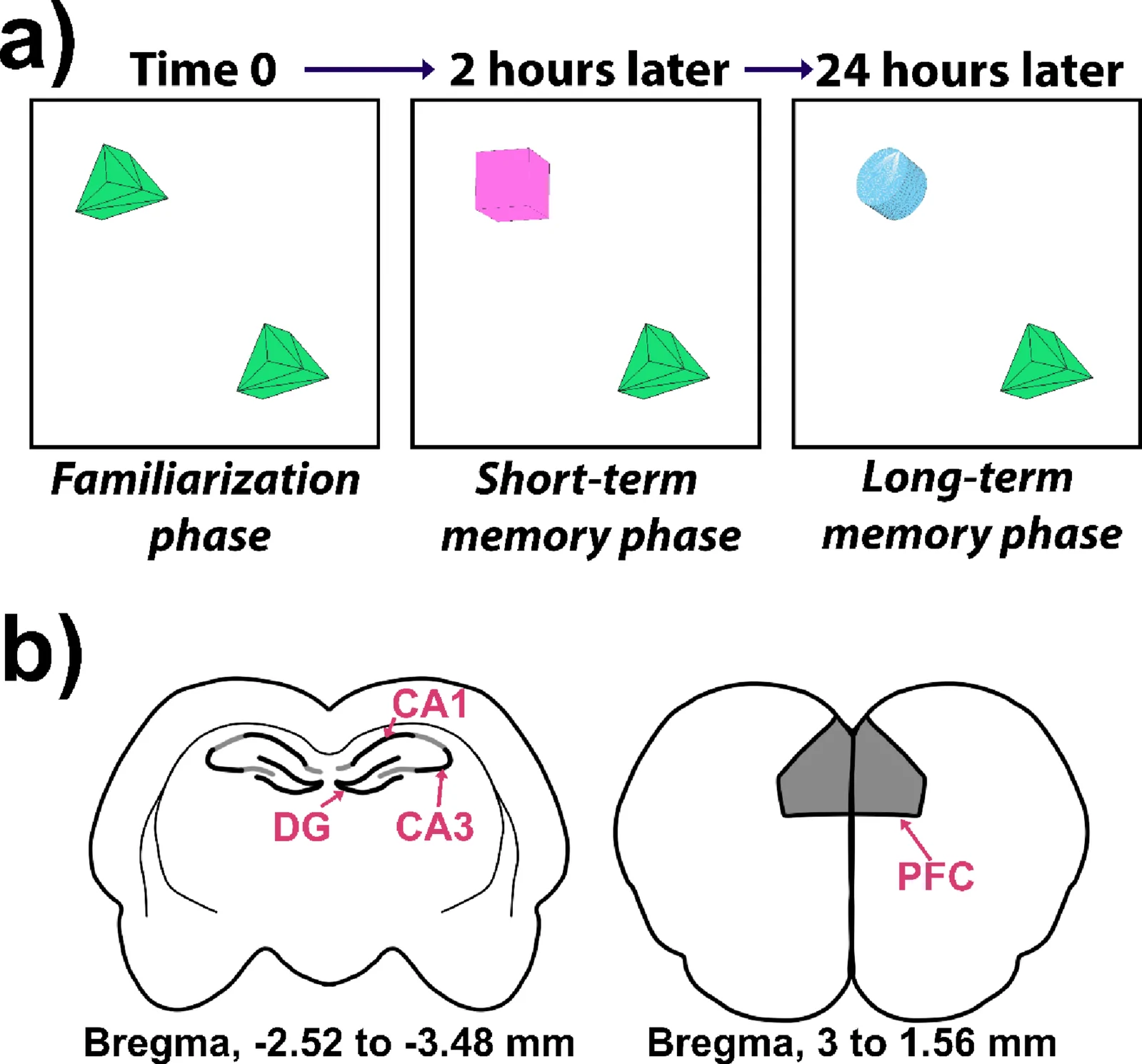
**a** Timeline for behavioral assessment. Following 12 weeks of treatment, behavioral assessments were performed after completion of the Phenylbutyrate administration period. Memory was evaluated using the Novel Object Recognition (NOR) test, a widely used paradigm to assess recognition memory. This procedure comprised three phases. During the familiarization (training) phase, rats were allowed to freely explore two identical objects within the testing arena. Memory retention was subsequently evaluated in short-term and long-term memory phases, during which one of the familiar objects was replaced with a novel object. Recognition memory was inferred from exploration behavior, as rat spent significantly more time interacting with the novel object than with the familiar object. **b** Corticolimbic regions analyzed in coronal planes. The CA1, CA3, and DG regions of the hippocampus were analyzed using slices at a distance of −2.52 to −3.48 mm from Bregma. Additionally, slices of the prefrontal cortex (PFC) were used at a distance of 3 to 1.56 mm from Bregma

Once PB or VH treatment was finished, animals were habituated and acclimated by placing them in the behavioral testing room 24 h before the tests begin. Rats were tested for recognition memory using a wood test box of 60 × 60 × 60 cm in a dark room with red light on 3 consecutive days as previously evaluated (Monroy et al. 2025) (Fig. 1a). The surfaces and walls of the testing box are painted, thereby reducing the wood’s porous, absorbent nature. Additionally, the box was cleaned (using Pinol^®^ household multi-purpose cleaner) and disinfected (with 70% ethanol) before introducing each rodent, thus preventing the presence of olfactory cues from previous rodents. Recognition memory was evaluated using the novel object recognition (NOR) test starting at day 2. This test was performed in three phases. *Familiarization phase*: Each rat was placed in the test box with 2 identical objects (familiar objects). *Short-term phase*: 2 h after the familiarization phase, the rats were placed again in the test box with one of the familiar objects and one new, novel object. *Long-term phase*: 24 h after, the rats were placed in the test box with one familiar object and a new novel object. Each phase lasted 6 min and the time spent exploring each object was counted for each rat. The objects were placed on the opposite side of the box, at the same distance between them and the box border as is represented in the Fig. 1a. The objects used were ceramic geometric prism (represented in the Fig. 1a) with similar size, same color (beige), and odor free. These were cleaned and sterilized before introducing each animal to avoid the odor interaction. Novel object Preference Index (PI) was calculated using the equation: PI= time exploring the novel object/time exploring the familiar object + time exploring the novel object. In addition, the time spent exploring the novel object and the familiar object was added together to estimate the total exploration time in each phase of the NOR test. All the tests were video recorded (Steren HD webcam, 1080p, 15/30 fps) for analysis. Only rats that successfully completed all three phases of the test were used. Object exploration was defined as only those instances where the rodent explored the objects using their whiskers and sense of smell. Behaviors such as climbing on the object, leaning against it, or making contact with body parts other than the nose and vibrissae were not scored as exploration (Lueptow 2017a). Furthermore, only rodents with a total exploration time of at least 10 s were considered.

### Tissue preparation

After behavioral testing was complete, rats were sacrificed using an overdose of pentobarbital (75 mg/kg i.p.). Once the rats were completely anesthetized and non-reflexive, approximately 4–5 min later, an intracardiac perfusion was performed. A transverse incision was made between the thorax and abdomen, as well as a longitudinal cut in the thorax to expose the heart. To ensure more efficient perfusion, the descending aorta was clipped, directing the entire perfusion solution only to the upper limbs and head. Subsequently, an incision was made in the right atrium to allow for blood drainage and perfusion. The perfusion was administered with 180 ml of physiological saline solution (Hartmann’s solution, PiSA Farmacéutica, Mexico) through the left ventricle at 20 ml/min (Aguilar-Hernández et al. 2020; Monroy et al. 2025). To confirm successful perfusion, a visual inspection of the thoracic blood vessels and aorta was performed. These should appear completely pale, with no blood present. Similarly, the eyes of the rat and the soles of its forefeet should appear completely white. Subsequently, the brain was obtained and immediately incubated in Golgi-Cox solution for 30 days and subsequently subjected to Golgi-cox staining method as detailed below and in previous publications (Boyzo Montes de Oca et al. 2024; Gibb and Kolb 1998; Monroy et al. 2025). After, the brains were incubated in sucrose solution (30%) for 7 days. Later, coronal Sect. (200 μm) were obtained using a manual vibratome (Leica) and placed on gelatinized slides. The sections/slides were subjected to the stain developing train in the following order: sodium hydroxide (25%) for 30 min, water for 5 min, kodak fixer (50%) for 30 min, water for 5 min, ethanol 70% for 5 min, ethanol 90% for 5 min, ethanol 100% 5 min x 2, and xylene for 15 min. Finally, slides were mounted using synthetic resin and a cover slip.

### Dendritic spine analysis

Once the slices were dry, they were observed using an optic microscope (Leica DMS100) adapted with a camera lucida (Aguilar-Hernández et al. 2020; Monroy et al. 2025). The regions of interest were localized using a rat brain atlas (Paxinos and Franklin 2019). Distal basal dendrites of pyramidal neurons from the CA1 and CA3 regions of the hippocampus (Bregma, −2.52 to −3.48 mm) and from layer 3 and 5 of the PFC (Bregma, 3 to 1.56 mm), and distal dendrites of granule neurons of the dentate gyrus (DG) (Bregma, −2.52 to −3.48 mm) were analyzed (Fig. 1b). For dendritic spine density, 10 segments of 30 μm of dendrite from different neurons by rodent was traced with their dendritic spines using the camera lucida at 1000X. Later, their spines were counted and the mean number of spines in 10 μm was determined. For dendritic spine morphology, the 100 most distal spines of these neurons were observed at 2000X and classified, according to publications (Pchitskaya and Bezprozvanny 2020) (Coatl-Cuaya, et al., 2022), based on their morphology in 5 categories: *mushroom* (M); short neck and big head, *thin* (T); large neck and small head, *stubby* (S); no-neck/head distinguishable, *bifurcated* (B); two heads, *unclassified* (U); other morphology. For both variables, spines density and spine morphology, 10 neurons from each region from each animal were analyzed. The values used in the statistical analysis were the mean of each rodent. These analyses were performed manually by experienced personnel with no connection to the project and were double-blind.

### Statistical analysis

All the data were analyzed using GraphPad Prism 10.6 software. Data were analyzed using unpaired Student’s t-tests. In addition to p values, Cohen’s d was calculated for each comparison as a measure of effect size. Additionally, a one sample t-test was used to analyze the PI of the NOR test using 0.5 as hypothetical value. In addition, a two-way ANOVA was used to analyze the total exploration time considering the treatment and NOR phases as the independent variables. The results in graphs are expressed as the mean ± the standard error of the mean. P values < 0.05 were considered as significant differences.

## Results

### Blood pressure measurements

**Fig. 2:**
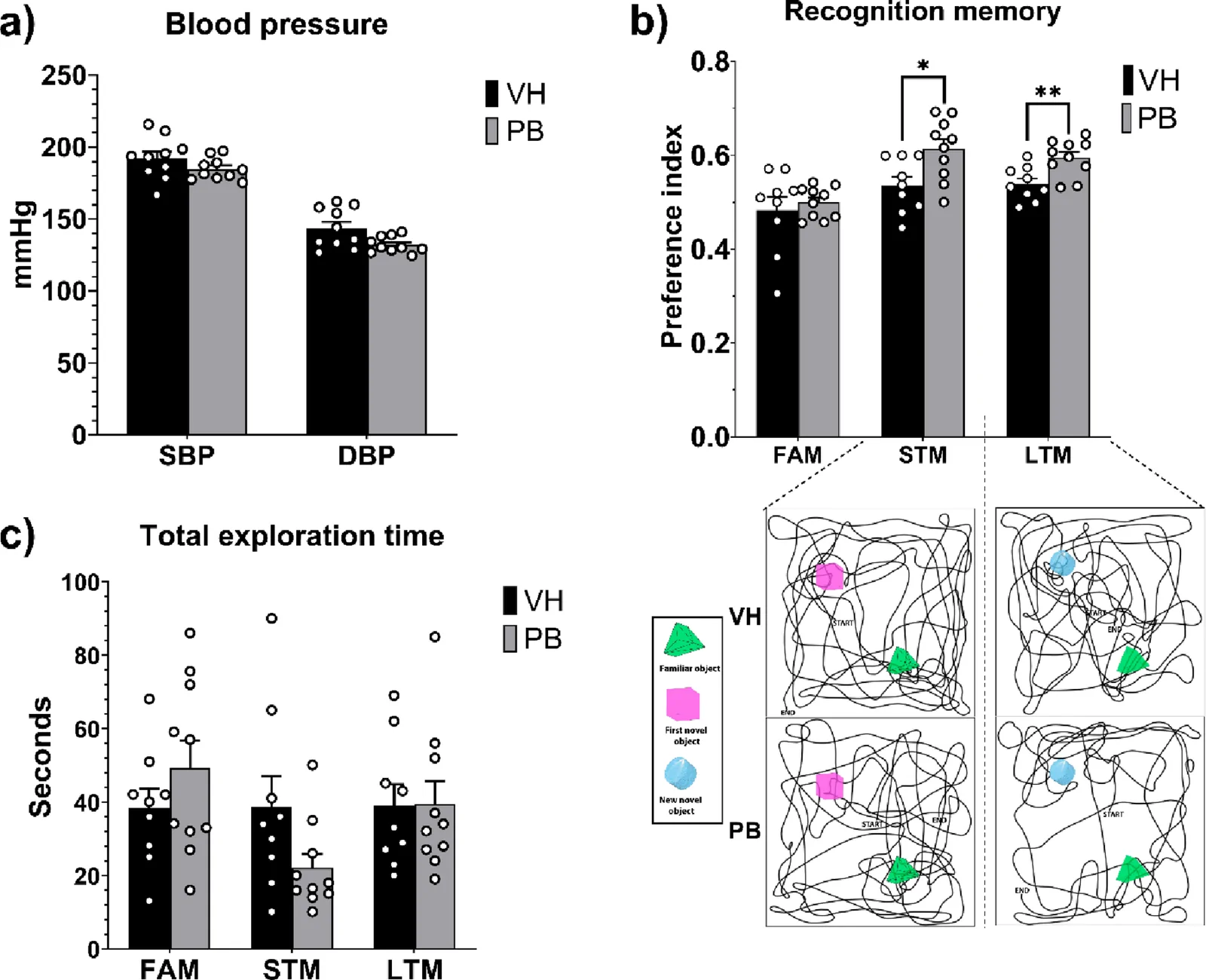
**a** Blood pressure estimation. Systolic and diastolic blood pressure measured in the caudal artery showed no differences when compared between the groups. The mean arterial pressure for the VH group was 192.28/143.76 mmHg, and for the PB group it was 184.77/132.23 mmHg. **b** Recognition memory in SH rats. Upper panel. Preference index for familiarization phase and short- and long-term memory, rats treated with PB have an increase in recognition memory. Lower panel. Representative performance for short- and long-term recognition memory in the NOR test. **c** Total time exploration. The exploration time for both objects in the NOR test was added together to determine the total exploration time. No differences were observed in the VH group in any of the phases of the NOR test. In the PB group, a significant reduction was observed in the STM phase compared to the FAM phase. No changes were observed when comparing the VH vs. PB groups. SBP: systolic blood pressure, DBP: diastolic blood pressure, STM: short-term memory, LTM: long-term memory, VH: control group, PB: phenylbutyrate group. *p < 0.05, **p < 0.01

BP was measured in each rat before the treatment (*n* = 10 by group). There are not significative changes in both systolic and diastolic BP between groups (Fig. 2a). The mean systolic BP in VH group was 192.28 ± 4.619 mmHg and 184.77 ± 2.407 mmHg for PB group. The diastolic BP was 143.76 ± 4.363 mmHg in VH group and 132.23 ± 1.776 mmHg for PB group. Notably, the BP measurements are typical of a SH rat (Solis-Gaspar et al. 2016).

### PB increases short- and long-term recognition memory

In the NOR test (*n* = 9 in VH group, *n* = 10 in PB group), we evaluated the ability of the rat to remember an object. In normal conditions, the rodents spend more time interacting with the novel object in comparison with the familiar object. If the rodents do not remember the familiar object, they will interact equally with both objects, which would result in a hypothetical PI of 0.5. In our results (Fig. 2b), there are no significant differences in the familiarization phase for any of the groups compared to the hypothetical value (0.5) (One sample t-test; VH p-value = 0.8240, PB p-value = 0.9687). In the same way, there were no significant differences in this phase compared between groups (t-test p-value = 0.8263, d = 0.11). In the short-term memory phase, a significantly higher PI was observed in the PB group (0.6133 ± 0.02) compared to the hypothetical value (One sample t-test; VH p-value = 0.0986, PB p-value = 0.0004). Furthermore, we observed that PB induces a significant increase in the PI compared to the control group (0.5357 ± 0.019) (t-test p-value = 0.0139, d = 1.26). Regarding long-term memory, it was observed that VH (0.539 ± 0.012) and PB (0.5946 ± 0.0123) groups had a significant increase in their PI compared to the hypothetical value (One sample t-test; VH p-value = 0.0117, PB p-value < 0.0001). The comparison between groups shows that rats treated with PB have a significantly higher PI compared to untreated rats (t-test p-value = 0.0050, d = 1.48).

Regarding the total exploration time, a two-way ANOVA revealed no significant main effects or interaction across any phases of the NOR test (Fig. 2c). During the FAM phase, rats treated with PB showed a non-significant increase in object exploration time. In the STM phase, the PB group exhibited a non-significant decrease in total exploration time compared to the VH group. Although a Tukey post-hoc test indicated a significant difference between the FAM and STM phases within the PB group (*p* = 0.0081), this result should be interpreted with caution given the absence of significant effects in the overall ANOVA.

### PB treatment modifies dendritic spines

**Fig. 3:**
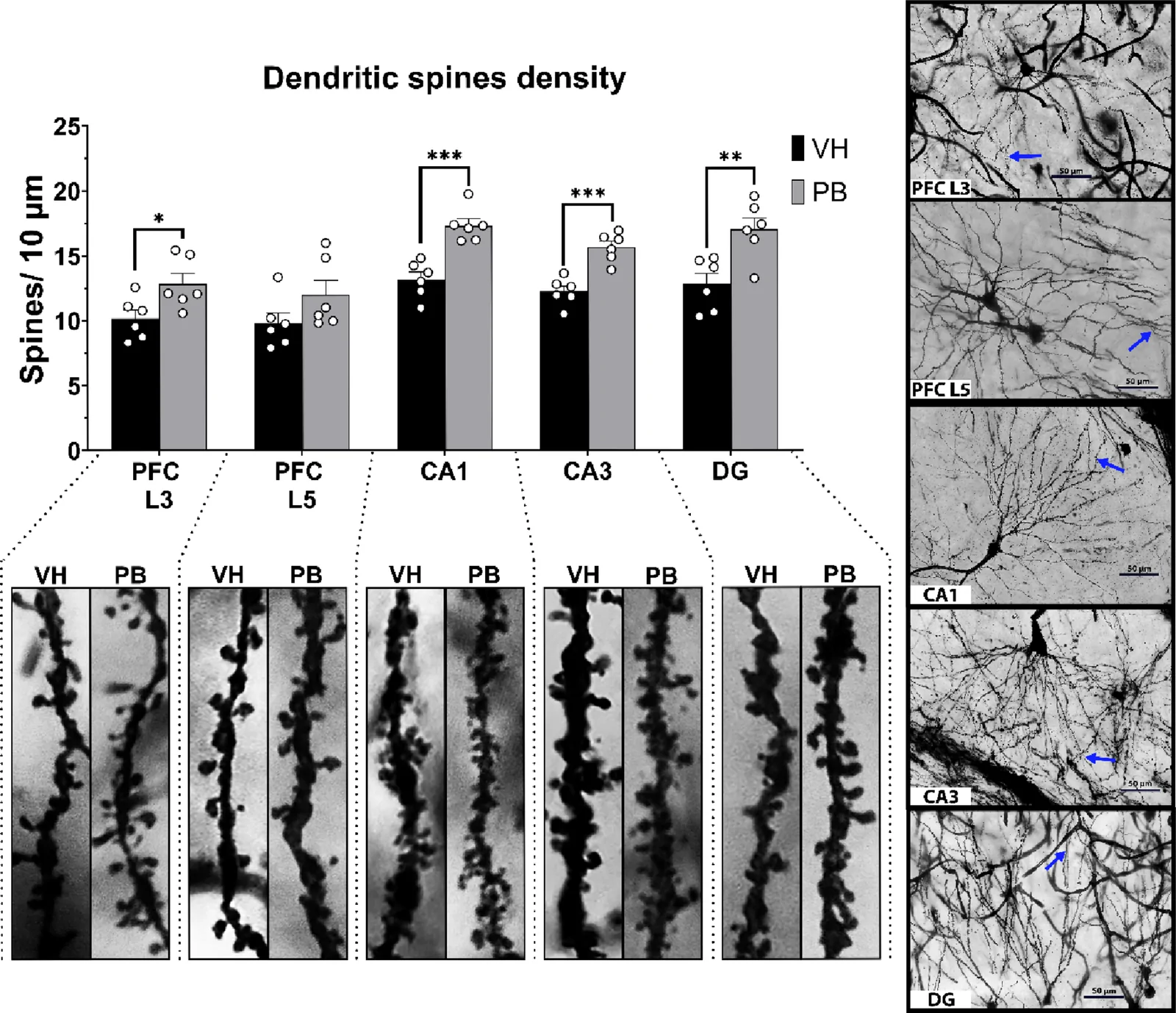
Dendritic spine density. Number of dendritic spines in 10 μm of distal dendrites. PB treatment increased spine density in all the analyzed regions except for neurons of the PFC L5. The right panel provides a photograph of a representative neuron from each region; a blue arrow indicates examples of dendrites used in the analysis. *PFC L3: prefrontal cortex layer 3*,* PFC L5: prefrontal cortex layer 5*,* DG: dentate gyrus*,* VH control group*, PB: phenylbutyrate group. *p < 0.05, **p < 0.01, ***p < 0.001

**Fig. 4:**
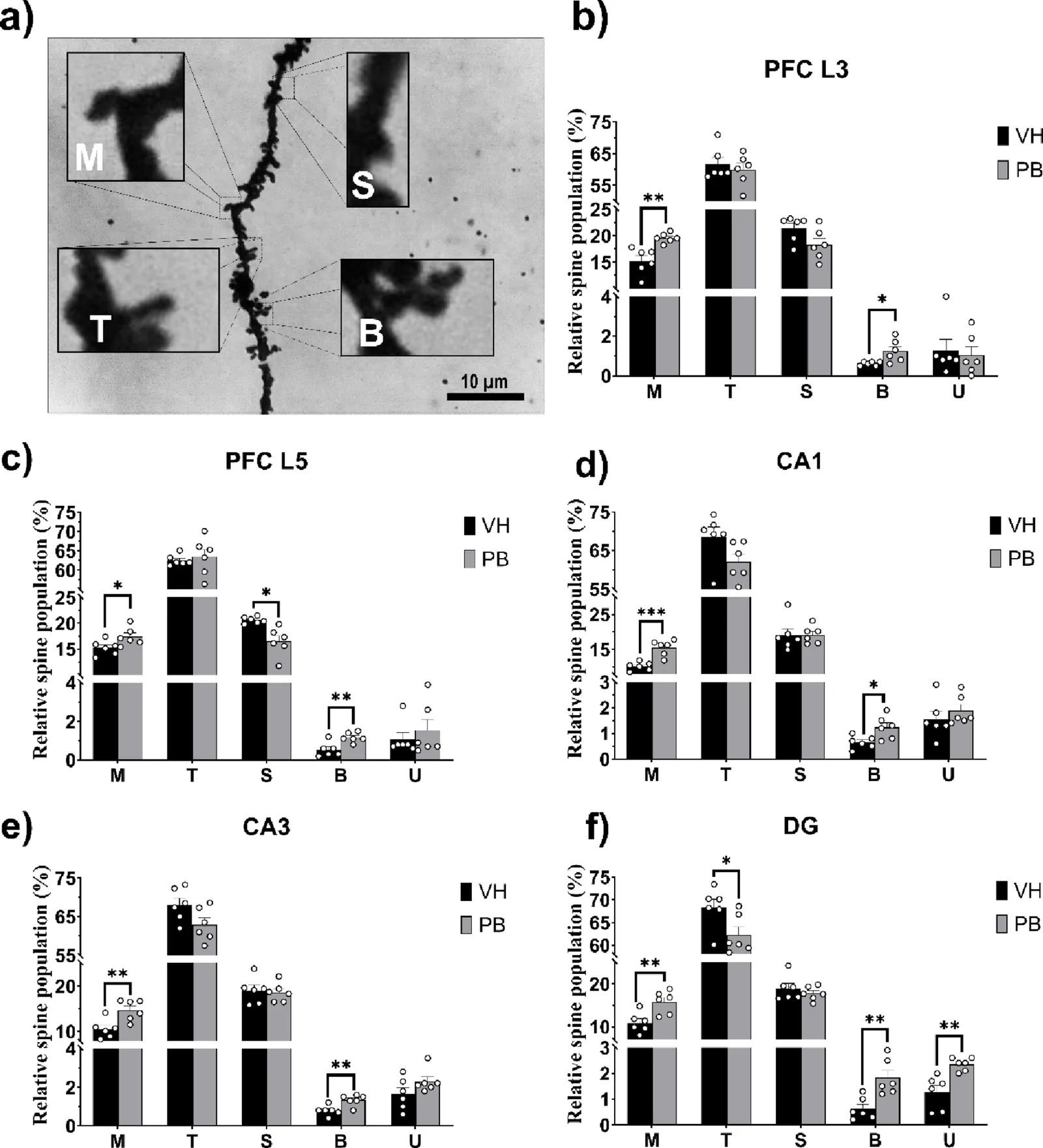
Dendritic spine morphology. **a** Representative distal dendrite with dendritic spine examples showing representative morphology. **b** Proportion of spine types in PFC L3. PB treatment increased M and B spines. **c** Proportion of spine types in PFC L5, PB increased M and B spines, and reduced S spines. **d** Proportion of spine types in CA1, PB increased M and B spines. **e** Proportion of spine types in CA3, PB increased M and B spines. **f** Proportion of spine types in DG, PB treatment increased M, B and U spines, and reduced T spines. *M: mushroom spines*,* T: thin spines*,* S: stubby spines*,* B: bifurcated spines*,* U: unclassified spines*,* PFC L3: prefrontal cortex layer 3*,* PFC L5: prefrontal cortex layer 5*,* DG: dentate gyrus*,* VH: control group*, PB: phenylbutyrate group. *p < 0.05, **p < 0.01.

For the dendritic spines analysis, we evaluate the density and the morphology of these structures in each one of the interest regions (*n* = 6 by group). PB treatment increases the density of dendritic spines (Fig. 3) in PFC layer 3 (t-test, *p* = 0.0296, d = 1.47, PB mean = 12.82 ± 0.8 vs. VH mean = 10.17 ± 0.65), hippocampus CA1 (t-test, *p* = 0.0003, d = 3.07, PB mean = 17.31 ± 0.53 vs. VH mean = 13.16 ± 0.57) and CA3 (t-test, *p* = 0.0003, d = 3.14, PB mean = 15.64 ± 0.46 vs. VH mean = 12.22 ± 0.43), and DG (t-test, *p* = 0.006, d = 2.00, PB mean = 17.0 ± 0.9 vs. VH mean = 12.77 ± 0.82) in comparison to the control group. Dendritic spines were classified and reported as percent of a population of 100 spines (Fig. 4). Mushroom spines are increased by PB treatment in all analyzed regions (t-test, PFC L3 *p* = 0.0062, d = 2.32; PFC L5 *p* = 0.045, d = 1.33; CA1 *p* = 0.001, d = 2.99; CA3 *p* = 0.0068, d = 1.97; DG *p* = 0.0072, d = 1.95) in comparison with the control group. PB treatment increased thin spines in the DG (t-test, *p* = 0.0389, d = 1.37) compared to the control group, with no changes found in PFC and hippocampus CA1/CA3. Stubby spines were reduced in pyramidal neurons of PFC L5 in the PB treated group (t-test, *p* = 0.0133, d = 2.07) compared to the control group, the rest of analyzed regions were not affected. Bifurcated spines were increased by the PB treatment in each analyzed region (t-test, PFC L3 *p* = 0.0408, d = 1.55; PFC L5 *p* = 0.0069, d = 2.03; CA1 *P* = 0.0235, d = 1.62; CA3 *p* = 0.0059, d = 2.02; DG *p* = 0.0064, d = 2.08). Unclassified spines showed an increase in neurons of the dentate gyrus in the treated group (t-test, *p* = 0.008, d = 2.18) compared to the control group, with no changes in the PFC and hippocampus CA1/CA3.

## Discussion

In this study, we found that PB treatment increased short- and long-term PI, indicating improved recognition memory. PB treatment was also associated with increased dendritic spine density in PFC layer 3, the hippocampal CA1 and CA3 regions, and the DG. Moreover, PB increased the density of mushroom and bifurcated spines across the analyzed regions. Thus, the improvement in recognition memory following PB treatment was accompanied by increased dendritic spine density and changes in spine morphology in the PFC and dorsal hippocampus. These findings suggest that the cognitive effects of PB may be related to structural remodeling of dendritic spines. However, correlation analyses and functional studies will be necessary to determine whether these structural changes are directly associated with the observed improvement in recognition memory.

### PB improves short- and long-term recognition memory performance

SH rats are characterized by spontaneously developing hypertension as shown in the PB measurements. Both age and high blood pressure have been associated with alterations in brain structure and cognitive processing (Pacholko and Iadecola 2024). The NOR test is based on the study of the natural behavior of rodents, evaluating the time they spend exploring a novel object versus a known object (Lueptow 2017b). Rodents tend to spend more time interacting with novel objects. Using this innate behavior, we can determine the rodent’s ability to remember an object with which it has already interacted (Leger et al. 2013). Our results show that PB improves short- and long-term recognition memory. Previously, other studies observed that PB also improves spatial orientation and memory (Carvajal-Flores et al. 2020; Yao et al. 2014), recognition memory and social interaction (Ryu et al. 2021) in rodents. Moreover, another study using sodium butyrate, a PB precursor, showed a recovery in the NOR test performance in animal models of autism (Takuma et al. 2014). In this study, locomotor activity was not independently assessed, therefore, we cannot completely exclude the possibility that PB may have influenced exploratory behavior and, consequently, performance in the NOR test. However, all animals met the predefined exploration criteria during the familiarization and testing phases, indicating adequate engagement with the task. Moreover, the primary outcome measure was the PI, which is based on the relative preference for the novel object and is independent on absolute levels of locomotor activity when sufficient object exploration is achieved (Antunes and Biala 2012; Ennaceur and Delacour 1988). Thus, the observed differences are more likely to reflect alterations in recognition memory than gross changes in motor function or exploratory drive. Nevertheless, future studies incorporating independent assessments of locomotion and anxiety-related behavior would further strengthen the interpretation of the cognitive effects of PB. Moreover, we used only the NOR test to measure cognitive function. Although this is an effective test for assessing memory, further cognitive testing is required to confirm that PB improves cognitive functions.

Learning is a complex cognitive process that requires the participation of limbicocortical regions such as the dorsal hippocampus, medial thalamus, entorhinal cortex, and PFC (Dubinsky and Hamid 2024). These connections are established at synapses through axon terminals and dendritic spines; more stable synapses underlie better learning and memory processes (Ma and Zuo 2022; Runge et al. 2020).

### PB treatment increases dendritic spine density and promotes a higher proportion of mushroom and bifurcated spines

Aging results in progressive loss of dendritic spines (Aguilar-Hernández et al. 2020; Monroy et al. 2025) as well as a decrease in spinogenesis in the hippocampus and cortex (Huang et al. 2020; Perez-Cruz et al. 2011). Meanwhile, previous reports in animal models of hypertension showed a reduction in dendritic spines in the PFC and hippocampus as well as memory deficits (Brocca et al. 2013; Sánchez et al. 2011; Vega et al. 2004). In our study, PB increased the number of dendritic spines in the PFC L3, dorsal hippocampus and DG. Indeed, PB increases dendritic spine density in the PFC, CA1 and nucleus Accumbens and dendritic length in CA1 and synaptophysin levels and reduces glial fibrillary acidic protein (GFAP), a marker of astrocytes, expression in aged mice (Carvajal-Flores et al. 2020). In animal models of Alzheimer’s disease, PB reduces the loss of dendritic spines (Ricobaraza et al. 2012). Furthermore, the use of PB precursors in animal models of autism restores dendritic spine density in the hippocampus (Takuma et al. 2014).

An increase in spine density does not necessarily indicate efficient neuronal communication, but rather that the morphology of spines has a closer relationship with the efficiency of synaptic transmission (Gipson and Olive 2017; Pchitskaya and Bezprozvanny 2020; Tendilla-Beltrán et al. 2019). Indeed, different types of dendritic spines have been described morphologically. Mushroom spines are generally associated with stronger and more stable synaptic transmission, whereas thin spines are typically smaller and more dynamic, and are often associated with less stable synaptic contacts and greater capacity for synaptic plasticity (Lee et al. 2024). Stubby spines display the lowest levels of synaptic transmission, while bifurcated spines are related with the formation and remodeling of new dendritic spines (Lynch et al. 2015; Tendilla-Beltrán et al. 2019; Trommald et al. 1996). In our research, we found PB treatment increased mushroom and bifurcated spine density in the PFC and dorsal hippocampus. Both types of spines are associated with improvements in synaptic transmission (Helm et al. 2021; Matsuzaki et al. 2001). Normally, a reduction in mushroom spines and an increase in stubby spines have been reported in aged rodents (Aguilar-Hernández et al. 2020; Dickstein et al. 2013; Monroy et al. 2025). In male animal models of hypertension, a reduction in dendritic length and dendritic spine density has been found in the hippocampus (Sabbatini et al. 2002), CA1, CPF, and nucleus Accumbens (Brocca et al. 2013; Flores et al., 2016). Thus, we found that PB improves the proportion of dendritic spine types.

Our results suggest that PB may mitigate age- and hypertension-associated alterations in dendritic spine density and morphology. Studies have reported that the different morphologies in dendritic spines have different proportions of proteins that are indispensable for the synapsis. Mushroom spines are rich in postsynaptic density protein 95 (PSD95), alpha-amino-3-hydroxy-5-methyl-4-isoxazolepropionic acid and N-methyl-D-aspartate receptors, actin and calmodulin (Arellano 2007; Bourne and Harris 2007; Lamprecht 2021; Priel et al. 2022). Reports have shown that the synaptic transmission in these spines is more efficient in comparison with the other spines (Araya 2022; Harnett et al. 2012). Considering this, our findings in dendritic spine density and morphology could be indirect evidence of synaptic efficacy. However, electrophysiological and molecular studies are required to a complete certainty about synaptic transmission.

Various studies have shown different mechanisms of action and properties of PB. It is reported that PB displays anti-inflammatory properties (Jangra et al., 2016; Roczkowsky et al. 2022) as it inactivates cytokines such as tumor necrosis factor-alpha, interferon-gamma and interleukin-6 (Hazekawa et al. 2018; Yadav et al. 2021), and inhibits the nuclear factor kappa-light-chain-enhancer of activated Bcells pathway (Koganti et al. 2022). Moreover, PB also reduces neuroinflammatory markers such as GFAP (Carvajal-Flores et al. 2020; Kasle et al. 2025), Ionized Calcium-Binding Adapter Molecule 1 (Iba1), a microglial marker (Huang et al., 2022) and inducible nitric oxyde synthase (Hazekawa et al. 2018; Qi et al. 2004). It has been observed that a chronic inflammatory state develops in aging (Soraci et al. 2024; Zhang et al. 2024) and in hypertension (Barrows et al. 2019; Krzemińska et al. 2022). The presence of pro-inflammatory cytokines has been linked to neuronal atrophy and cognitive deficits (Liang et al. 2024; Luo et al. 2022). On the other hand, some studies show that PB have antioxidant properties (Jangra et al., 2016; Yang et al. 2016) by reducing reactive oxygen species (Jangra et al. 2016; Luo et al. 2010). Oxidative stress is one of the typical characteristics of the neuronal environment in aging (Maldonado et al. 2023; Warraich et al. 2020) and hypertension (Griendling et al. 2021; Touyz et al. 2020). Furthermore, oxidative stress is also associated with cognitive impairments (Franzoni et al. 2021; Logan et al. 2019). Likewise, it has been proposed that the main mechanism of action of PB is the inhibition of histone deacetylase (Faillace and Bernabeu 2019; Iannitti and Palmieri 2011; Spartalis et al., 2019), an enzyme that regulates the access of the transcription machinery to DNA by removing acetyl groups from histones (Park and Kim 2020; Porter and Christianson 2019).

In chronic inflammatory conditions in the brain, cellular dysfunction and apoptosis appear (Tang et al. 2022). In hypertension, there is a modification of hemodynamic stress, an alteration of laminar flow, and an adverse mechanical environment in the cells, which favors the activation of cellular stress pathways, including endoplasmic reticulum (ER) stress (Camargo et al. 2023; Kassan et al. 2012; Young 2017). These alterations result in cellular dysfunction that mainly affects endothelial cells and neurons, influencing brain atrophy and cognitive functions (Ekundayo et al. 2024; Perner and Krüger 2022). Studies propose that the beneficial effects of PB are also mediated by its ability to alleviate ER stress, leading to restored eNOS signaling, reduced oxidative stress, and attenuation of inflammation and apoptosis (Ma et al. 2025; Pao et al. 2021). Several reports have shown that PB is capable of reducing the ER stress in animal models of renal fibrosis (Liu et al. 2016), retinal damage (Huang et al., 2022), Dravet syndrome (Shen et al. 2024), diabetes (Srinivasan and Sharma 2011), ischemic brain injury (Wu et al. 2021), spinal cord injury (Zhou et al. 2016), human cell culture (Kubota et al. 2006; Mimori et al. 2012), and in viral infection (Wang et al. 2023).

The histone deacetylase (HDAC) inhibitory activity of PB promotes a chromatin state permissive to transcription (Juarez et al. 2024). This property is particularly relevant in the context of aging and hypertension, conditions associated with alterations in epigenetic homeostasis, increased HDAC activity, neuroinflammation, and impaired expression of genes involved in synaptic plasticity and cognitive function (Ding et al. 2023; Zocher 2024). Reports have shown that histone deacetylase inhibition promotes dendritic growth, increase dendritic spine density, and improve learning and memory (Fischer et al. 2007; Kitahara et al. 2021; Wong et al. 2020).

Interestingly, the epigenetic actions of PB may also interact with estrogen-dependent signaling. Estrogens exert neuroprotective and procognitive actions through estrogen receptors which function as ligand-activated transcription factors (Arevalo et al. 2015). Upon activation, these receptors recruit coactivator complexes possessing histone acetyltransferase activity, including CBP/p300 and members of the steroid receptor coactivator family (Maioli et al. 2021; Toumba et al. 2024), thereby increasing histone acetylation and enhanced transcription of genes involved in neuronal survival, synaptic plasticity, and memory formation (Frick 2015). Consequently, estrogen signaling contributes to the maintenance of a permissive epigenetic environment that supports dendritic spine stability and optimal cognitive performance (Hara et al. 2015; Sheppard et al. 2019). This interaction may be particularly relevant in the female animals examined in the present study. By 12 months of age, female rats may exhibit age-related alterations in ovarian hormone signaling that may compromise estrogen-mediated transcriptional regulation (de Assis et al. 2025; Frankfurt et al. 2023). Reduced estrogenic signaling has been associated with decreased histone acetylation, diminished expression of plasticity-related genes such as BDNF, Arc, and PSD-95, and impairments in hippocampal-dependent memory processes (Beamish et al. 2026; Luine et al. 2018). Consequently, the decline in estrogen-dependent epigenetic regulation during aging may contribute to synaptic dysfunction and cognitive deficits. Increased histone acetylation may facilitate the transcription of genes involved in synaptic remodeling, dendritic spine maintenance, and long-term memory consolidation (Burns and Gräff 2021; Gräff and Tsai 2013). Such mechanisms could contribute to the preservation of dendritic spine populations observed in the hippocampus and PFC, regions critically involved in recognition memory and executive cognitive processes (Chao et al. 2022; Lissner et al. 2021; Wang et al. 2021). However, future studies are required to directly assess the effects of PB-mediated epigenetic modulation and estrogen-mediated epigenetic pathways to determine whether these mechanisms contribute to the preservation of synaptic architecture and cognitive function in aging hypertensive females.

Several limitations should be considered when interpreting our findings. First, we did not assess molecular or cellular markers directly associated with the mechanisms of action of PB. Therefore, although our findings are consistent with potential anti-inflammatory, antioxidant, and epigenetic effects of PB, it is not possible to determine this from our data. Second, cognitive assessment was limited to the NOR test, therefore, whether the beneficial effects of PB extend to other cognitive domains remains to be determined using complementary behavioral paradigms. Third, dendritic spine density and morphology were evaluated as structural correlates of synaptic plasticity, but synaptic function was not directly assessed. Thus, the observed structural changes should not be interpreted as direct evidence of enhanced synaptic transmission. Moreover, the cross-sectional design and the use of a single age group prevent determining whether PB prevents, reverses, or otherwise modifies age-related cognitive and structural alterations. Finally, because only female rats were included, the generalizability of these findings to males remains to be established.

In conclusion, PB treatment improved recognition memory and increased dendritic spine density in the PFC and hippocampus, with a selective enrichment of mushroom and bifurcated spines. Our findings suggest that PB may mitigate age- and hypertension-associated cognitive and structural alterations in female SH rats, potentially by promoting dendritic spine maintenance and remodeling.

## Data Availability

All raw data generated and analyzed for this article are not publicly available due to intellectual property reasons. But they are available through the corresponding author upon reasonable request.
